# Animal reactivity to camera traps and its effects on abundance estimate using distance sampling in the Taï National Park, Côte d’Ivoire

**DOI:** 10.7717/peerj.13510

**Published:** 2022-05-27

**Authors:** Noël Adiko Houa, Noémie Cappelle, Eloi Anderson Bitty, Emmanuelle Normand, Yves Aka Kablan, Christophe Boesch

**Affiliations:** 1 Unité de Formation et de Recherches Biosciences, Université Felix Houphouët-Boigny, Abidjan, Côte d’Ivoire; 2 Wild Chimpanzee Foundation, Abidjan, Côte d’Ivoire; 3 Centre Suisse de Recherches Scientifiques, Abidjan, Côte d’Ivoire; 4 Department of Primatology, Max Planck Institute for Evolutionary Anthropology, Leipzig, Germany

**Keywords:** Abundance estimation, Camera trap distance sampling, Multi-species, Reaction, Species-specific ethogram, Taï National Park, Visual and olfaction signals

## Abstract

The use of camera traps (CTs) has become an increasingly popular method of studying wildlife, as CTs are able to detect rare, nocturnal, and elusive species in remote and difficult-to-access areas. It thus makes them suited to estimate animal density and abundance, identify activity patterns and new behaviours of animals. However, animals can react when they see the CTs and this can lead to bias in the animal population estimates. While CTs may provide many advantages, an improved understanding of their impacts on individual’s behaviour is necessary to avoid erroneous density estimates. Yet, the impact of CTs on detected individuals, such as human odour near the device and the environment, or the infrared illumination, has received relatively little attention. To date, there is no clear procedure to remove this potential bias. Here, we use camera trap distance sampling (CTDS) to (1) quantify the bias resulting from the different animal responses to the CTs when determining animal density and abundance, and (2) test if olfactory, visual and auditory signals have an influence on the animals’ reaction to CTs. Between March 2019 and March 2020, we deployed CTs at 267 locations distributed systematically over the entire Taï National Park. We obtained 58,947 videos from which we analysed four medium- to-large-bodied species (Maxwell’s duiker (*Philantomba maxwellii*), Jentink’s duiker (*Cephalophus jentinki*), pygmy hippopotamus (*Choeropsis liberiensis*) and Western chimpanzee (*Pan troglodytes verus*)) displaying different behaviours towards the CTs. We then established species-specific ethograms describing the behavioural responses to the CTs. Using these species-specific responses, we observed that the Maxwell’s duiker reacted weakly to CTs (about 0.11% of the distance data), contrary to Jentink’s duiker, pygmy hippopotamus and Western chimpanzee which reacted with relatively high frequencies, representing 32.82%, 52.96% and 16.14% of the distance data, respectively. Not taking into account the species-specific responses to the CTs can lead to an artificial doubling or tripling of the populations’ sizes. All species reacted more to the CTs at close distances. Besides, the Jentink’s duiker and the pygmy hippopotamus reacted significantly more to the CTs at night than during the day. Finally, as for olfactory signals, the probability of reaction to the CTs during the first days after CTs installation was weak in Maxwell’s duiker, but concerned 18% of the video captures in Western chimpanzees which decreasing with time, but they remained high in pygmy hippopotamus and Jentink’s duiker (65% and 70% of the video captures respectively). Careful consideration should be given to animal’s response to CTs during the analysis and in the field, by reducing human’s impact around the CTs installation.

## Introduction

Unbiased estimates of species density and abundance are critical to understand population evolutionary processes in order to assess and select conservation management actions (Rowcliffe et al., 2011; Crum, Neyman & Gowan, 2021; Amburgey et al., 2021).

To assess animal density in the wild, camera traps (CTs) became a popular tool to monitor species in the wild, because they are very efficient at recording continuously quantitative observations for elusive, nocturnal and cryptic species in remote habitats without human presence (Burton et al., 2015; Rovero & Zimmermann, 2016). As a result, several methods to estimate animal abundance were developed in the past years. The spatially-explicit capture-recapture (SECR) approach is up to now the most-developed method that allows researchers to estimate the abundance of individually identifiable species, such as tigers or chimpanzees (Chandler & Andrew Royle, 2013; Després-Einspenner et al., 2017). However, most of the species in the wild cannot be identified individually. To overcome this problem, new methods have been developed targeting a wider range of species, namely the ‘random encounter’ model (REM; Rowcliffe et al., 2008) based on models predicting collision rates in an ideal gas (Hutchinson & Waser, 2007). This means that an estimator for animal density can be derived from the rates of contact between animals and CTs (Rowcliffe et al., 2008). The REM requires accurate estimates of day range as well as the speed of animal movement, which may be difficult to obtain or to estimate precisely. Thus, an extension of the REM, the ‘random encounter and staying time’ model (REST; Nakashima, Fukasawa & Samejima, 2018) was developed. The REST model describes the relationship among staying time, trapping rate, and density, which is estimable using a frequentist or Bayesian approach. Similarly, Moeller, Lukacs & Horne (2018) developed three novel methods to estimate abundance of unmarked animals: a ‘time to event’ model to estimate abundance from trapping rate; a ‘space to event’ model that is not sensitive to movement rate and an ‘instantaneous sampling estimator’ that applies fixed-area counts to CTs.

All these proposed estimators have had little evaluation or testing in the field (Moeller, Lukacs & Horne, 2018; Nakashima, Fukasawa & Samejima, 2018).

Camera trap distance sampling (CTDS) contrary to other approaches has received validation in the field (Howe et al., 2017; Cappelle et al., 2019; Harris et al., 2020). CTDS considers CTs as observers in lieu of humans and accounts for animal movement by recording observation distances at predefined snapshot moments. It relies on the usual assumptions of distance sampling that (1) lines or points are placed independently of the distribution of animals, (2) animals on the line or point are detected with certainty, (3) animals are detected at their initial location prior to any movement in response to the observer and (4) animals do not react to the CTs (*i.e.*, move independently to the CTs) (Buckland et al., 2001; Howe et al., 2017).

Following the CTDS method, each position of an animal at a predetermined snapshot interval is considered as an independent observation (*e.g.*, every 2s; Howe et al., 2017). However, the use of multiple detections of the same animal via the snapshots approach violates the assumption that detections are independent events and therefore affects the variance of the estimate and the method to select the detection function model (Howe et al., 2017). We can relax this assumption by estimating variances using a nonparametric bootstrap, resampling points with replacement (Buckland et al., 2001) and following a new approach for model selection (Howe et al., 2019).

Yet, several previous studies showed that animals may react to human observers and CTs, which violates the last assumption (Buckland et al., 2001; Rovero & Marshall, 2004; Buckland et al., 2010; Kalan et al., 2019; Bessone et al., 2020). Therefore, these reactions can bias negatively or positively density estimates depending on the type of behaviour elicited (attraction or avoidance) (Buckland et al., 2001; Howe et al., 2017; Bessone et al., 2020; Cappelle et al., 2021). Animals can react to CTs in different ways (Kalan et al., 2019). For example, primates, with binocular vision, were more likely to detect and, hence, respond to devices when they were facing and traveling toward the CTs. Bonobos and gorillas had stronger looking impulses compared to chimpanzees, and reactions depended on social and environmental factors.

The bias created by animals reacting to the CTs is important. If animals avoid the field of view of the CTs or remain less in front of the CTs than expected, it can lead to an underestimation of the population size or density of that species. Conversely, animals that stay longer in the detection zone leads to an overestimation of the population size or density. To overcome this problem, it was proposed to discard the first period of the survey (Howe et al., 2017) or to exclude all the observations where animal behaviour obviously indicated a reaction to the CTs (Bessone et al., 2020; Cappelle et al., 2021). None of these proposed approaches totally correct the bias induced by reactive animals, and no clear procedure helps to solve this potential violation (Palencia et al., 2021). Hence, it is necessary to provide an effective and efficient approach to deal with animal’s behaviour toward CTs to estimate animal density without bias.

In this study, we applied CTDS to multiple species at a large spatial scale (5,360 km^2^) to (1) describe and quantify animal reaction to CTs by using species-specific ethograms; (2) assess the bias induced by reactive behaviour to CTDS density estimates; (3) test if olfactory, visual and auditory signals influenced animal reactivity to CTs.

## Materials & Methods

### Study area

**Figure 1:**
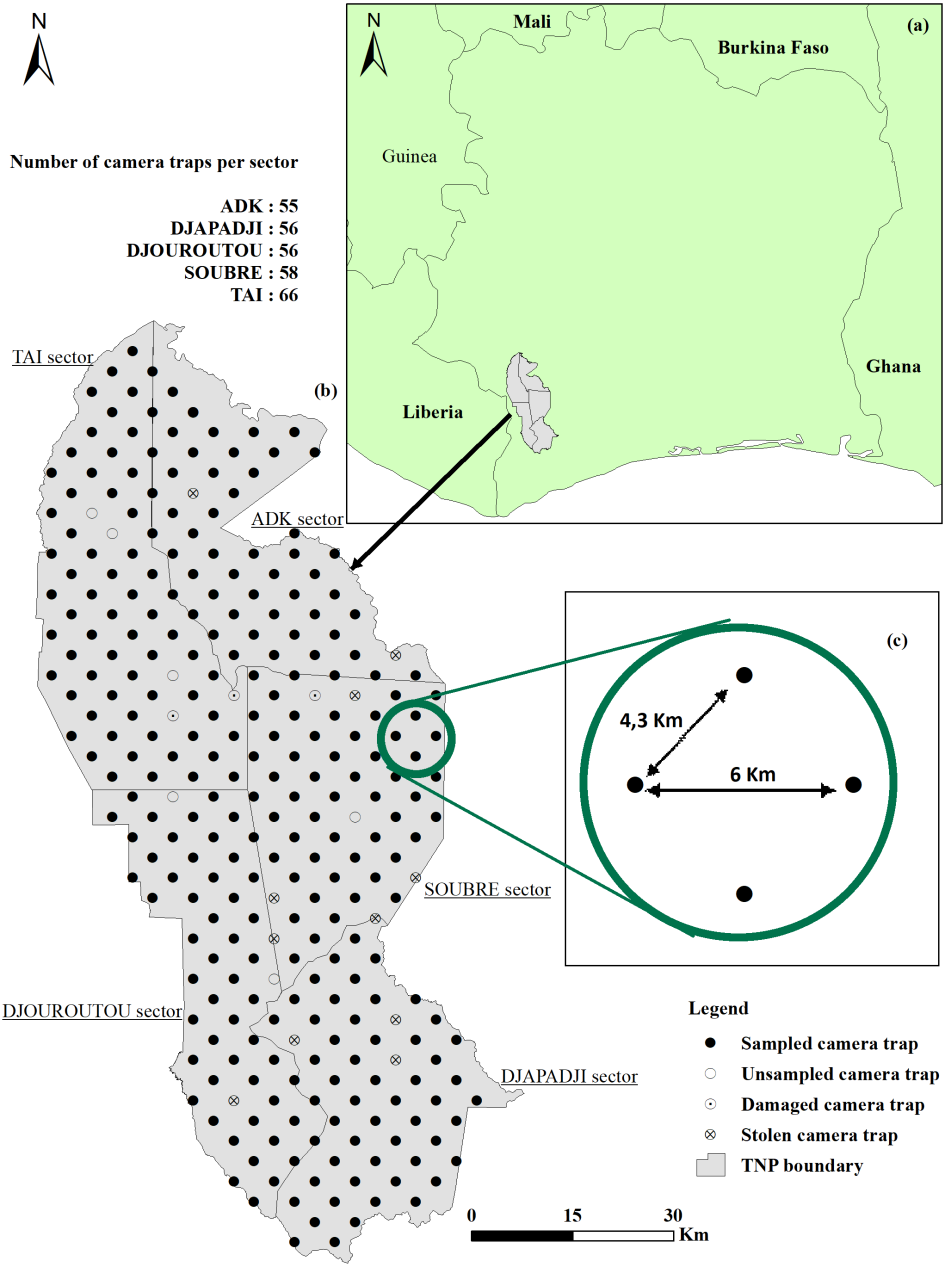
Location and survey design. (A) Côte d’Ivoire boundary and Taï National Park (TNP) location; (B) study area and location of cameras (C) distance (km) between camera traps.

We conducted the survey in the Taï National Park (TNP), covering an area of 5,360 km^2^, including the N’Zo Partial Faunal Reserve (PAG-PNT, 2020-2029). The TNP is located in the southwest of Côte d’Ivoire (Fig. 1A). The TNP was listed in the world network of Biosphere Reserves in 1978 and recognized as a World Heritage of the UNESCO since 1982. In addition, the TNP is one of the largest remaining and undisturbed lowland rainforests in West Africa (Lauginie, 2007). The average annual rainfall with the park is of 1,800 mm with an average annual temperature which swings between 24 °C and 30 °C (Anderson et al., 2005).

The large biodiversity observed in the TNP includes many endemic and threatened species. The park contains at least 1,366 plant species among which 25% are endemics (Scouppe, 2011). The northern part is mainly covered by *Eremospatha macrocarpa* and *Diospyros mannittiera* trees while the southwest includes water-demanding species such as *Diospyros spp.* and *Mapania spp* (Kolongo et al., 2006). About 145 mammal species were identified, among which the African forest elephant, *Loxodonta cyclotis* (Matschie, 1900), the Jentink’s duiker, *Cephalophus jentinki* (Thomas, 1892), the Zebra duiker, *Cephalophus zebra* (Gray, 1838), the Diana monkey, *Cercopithecus diana* (Linnaeus, 1758) and the pygmy hippopotamus, *Choeropsis liberiensis* (Morton, 1849) (Hoppe-Dominik, 1995; Chatelain et al., 2001). Moreover, 234 bird species are known in the TNP, including the White-breasted Guineafowl, *Agelastes meleagrides* (Bonaparte, 1850), a species of high conservation interest (Gartshore, Taylor & Francis, 1995; Fishpool, 2001). In addition, the TNP also holds 56 species of amphibians, 42 species of reptiles and 60 species of fish divided into 20 families and 37 genera inventoried in 2012 (Rödel & Ernst, 2004; Grell, Thiessen & Kouamelan, 2013).

### Data collection

Data were collected from March 2019 to March 2020 in the TNP with the approval from the Office Ivoirien des Parcs et Réserves (OIPR). The infrared CTs (Bushnell Trophy Cam HD Aggressor, Model 119877) used, were installed on trees (with a Diameter Breast Height (DBH) of about 10 cm) within a 30 m radius of the design-specified locations at a height of 50 cm above-ground, heading toward the geographical north (0° ± 20°) to avoid backlight (Howe et al., 2017; Apps & McNutt, 2018; Cappelle et al., 2021). CTs have a field of view *θ* = 38° and the trigger delay was set to minimum (0.6 s).

Given the size of the TNP, the study area was divided into sectors (Fig. 1B), according to the administration limits defined by the OIPR, the Ivoirian parks and reserves authority: ADK-V6: 1,020 km^2^; Djapadji: 1,020 km^2^; Djouroutou: 980 km^2^; Soubré: 1,040 km^2^ and Taï: 1,300 km^2^. In each sector, two teams (10 teams in total) installed and collected 40 CTs Bushnell Trophy Cam HD Aggressor, totalling 200 CTs used in this study. We surveyed 291 locations systematically placed 4.3 km apart for the shortest distance and 6 km for the farthest (Fig. 1C). One CT was set up on each point transect and stayed for a minimum of three months (average = 102 days, SD = 8.31) on the same location, before being relocated to a new one until covering all points in the five sectors. Out of the 291 points, 24 did not provide usable data: six were not surveyed due to their proximity to rivers or to logistical and field constraints, 15 CTs were destroyed or stolen by poachers and three CTs stopped working after two days because they recorded leaves facing the CTs. Consequently, the data were collected on only 267 point transect instead of 291. We programmed the CTs to stay active 24 h/day and to record 60s-long videos when triggered. When the CT was set, reference videos of researchers holding signs indicating distances from the CT (from 1 to 15 m at 1-m intervals, in the centre, and at each side of the CT’s field of view) were recorded at each location so that we could subsequently measure distances between the animals and the CT (see Howe et al., 2017 for details). Time of installation, habitat type, presence of rivers and fruits, Global Positioning System (GPS) location, Secure Digital (SD) card number and CT name were recorded as well for each device.

### Measurements and video analysis

After processing all videos (*i.e.*, to record the species and count the number of individuals detected, along with location, date and time of recording), we selected four medium-to-large-bodied species that displayed a range of behaviour toward the CTs (from none to many and with different way of displaying) for our analysis: the Maxwell’s duiker which is least concerned (LC), the Jentink’s duiker and the pygmy hippopotamus which are Endangered (EN), and the Western chimpanzee which is critically endangered (CR) (IUCN, 2021).

Preliminary viewing of the Jentink’s duikers and the pygmy hippopotamus videos revealed that they obviously reacted to the CTs as well as spending long time very close to them, without obvious reactions. Thanks to meticulous analyses, we pointed out other reactions to the CTs such as animals sniffing the CT. Thereafter, we identified all the reactions to the CTs device for the four species and established precise ethograms to distinguish species-specific reactions to the CTs (Table 1).

**Table 1:** Species-specific ethogram for determination of the proportion of reactions to the camera-trap device via video clips of Maxwell’s duiker, Jentink’s duiker, pygmy hippopotamus and Western chimpanzee.

Behaviour type	Behaviour	Description
Normal behaviour	Moving^M,C,J,H^	Animal follows a trajectory
		Sitting down at camera^C^
	Feeding^M,C,J,H^	Animal moves slowly, head down and sniffing, stopping to eat or to chew
		Animal foraging
	Grooming^M,C,J^	Animal licks or scratches itself
Reaction to camera	Attraction/fixation^M,C,J,H^	Animal looks directly at camera and moves towards it
		Sudden cessation of normal behaviour after looking in the direction of the camera
		Animal remains motionless and stares at the camera
		Animal touches camera after looking at it
		Animal touches or sniffs the camera
	Avoidance/running away^M,C,J,H^	Animal moves slowly/quickly after looking for camera either following or changing trajectory
		Animal appears surprised by camera and jumps back slightly, interrupting their current activity, either following or changing trajectory
		Animal moves away from camera after looking it
	Inspection^C,J,H^	Static or moving very slowly and sniffing in all directions
		Animal pausing and sniffing around and mainly towards the camera
		Animal moves slowly head down and ends up in front of the camera
		Animal sniffs branches cut or touched by field teams in front of the camera

**Notes.**

Particularities: •Do not record distances when the reaction to camera modified the trajectory of the animals. •If the animals remain in front of the camera after an apparent reaction, but head cannot be seen, probably the animal is still inspecting the camera.

M Maxwell’s duiker C chimpanzee J Jentink’s duiker H pygmy hippopotamus

We measured distances between CTs and the individuals of four species cited above at predetermined snapshot moments two seconds apart (at 0, 2, 4, . . . , 58 s after the even minute) as previously done (Howe et al., 2017; Bessone et al., 2020; Cappelle et al., 2019; Cappelle et al., 2021). To do so, the animal and the reference videos were compared to measure the distance between the animal and the CTs. As longer distances were more difficult to measure precisely than closer distances to the CTs, videos with animals more than 8 m from the CTs were assigned to one of the following categories: 8–10 m, 10–12 m, 12–15 m. At the same time, we also recorded the behaviour of all the animals, based on our ethogram.

### Quantifying the bias in abundance estimation

To evaluate the bias resulting from fully or partially ignoring animal response to the CTs, we proceeded in three successive steps, (first) we estimate population size using all video clips of an animal species; then (second) we excluded the videos presenting obvious responses to the CTs (like was done by Bessone et al., 2020), and finally we excluded all videos presenting species-specific reactions to the CT following our ethogram (Table 2).

**Table 2:** Details of the top three analysis performed for the four species.

Species	Analysis	Individual locations considered	Videos Considered	Radial distances	Selected model	Bootstrap CV%	Density (ind./km^2^)			Abundance		
							**Estimate; SE**	**95% CIs**		**Estimate; SE**	**95% CIs**	
Maxwell’s duiker	(1)	184	2263	13,206	Uni1	10	21.19; 2.41	17.79	25.00	113,604; 12,923.93	91,247.69	131,673.7
	(2)	184	2263	13,192	Uni1	10	21.17; 2.41	17.73	24.60	113,468; 12,915.94	92,416.57	137,122.1
	(3)	184	2263	13,192	Uni1	10	21.17; 2.41	17.73	24.6	113,468; 12,915.94	92,416.57	137,122.1
Jentink’s duiker	(1)	128	514	3,648	Hn0	14	0.75; 0.10	0.55	0.95	4,007; 510.53	2,814.01	5,195.18
	(2)	128	511	2,946	Hn1	14	0.68; 0.09	0.51	0.87	3,654; 472.13	2,791.19	4,687.59
	(3)	120	389	2,450	Hr0	18	0.26; 0.04	0.19	0.37	1,389; 196.75	690.52	1,328.74
Pygmy hippopotamus	(1)	78	257	2,587	Hr0	22	0.81; 0.63	0.55	1.20	4,361; 3,392.79	2,790.92	6,360.31
	(2)	76	233	2,165	Hr0	25	0.59; 0.56	0.31	0.92	3,147; 3,005.66	2,000.22	4,944.20
	(3)	72	188	1,217	Uni2	26	0.23; 0.07	0.14	0.35	1,255; 377.75	731.39	1,763.98
Western chimpanzee	(1)	65	211	2,896	Hn1	30	0.38; 0.13	0.21	0.60	2,012; 687.89	1,073.85	3,267.88
	(2)	63	197	2,455	Hr0	39	0.26; 0.09	0.09	0.52	1,384; 505.06	684.48	2,661.60
	(3)	61	184	2,421	Hr0	43	0.23; 0.09	0.09	0.45	1,236; 478.65	472.03	2,504.6

**Notes.**

Hn0 half-normal key function Hn1 half-normal key function with Hermite (4) adjustments Hr0 hazard-rate key function Uni1 uniform key function with cosine (1) adjustments

All densities were estimated by applying the following formula (Howe et al., 2017):

\begin{eqnarray*}\hat {D}= \frac{\sum _{k=1}^{K}{n}_{k}}{\pi {w}^{2}\sum _{k=1}^{K}{e}_{k}{\hat {P}}_{k}} \ast \frac{1}{A} \end{eqnarray*}

where “*n*_*k*_” is the number of observations of distance between animals and CT “k”, “w” is the maximum distance between objects and CT or truncation distance, “${\hat {P}}_{k}$” is the estimated probability of an image of an animal that is within “*θ*” and “w” front of the CT at a snapshot moment and “${e}_{k}= \frac{\theta {T}_{k}}{2\pi t} $” is the sampling effort at CT location “k”. Here, “*T*_*k*_” is the sampling effort at CT “k” in seconds (all operational days, excluding days of CT installation and retrieval, were considered when calculating survey effort), “t” is the time interval of predetermined snapshots (set to 2 s), “*θ*” is the horizontal viewing angle of the CT in radians (*i.e.*, 0.663), so “$ \frac{\theta }{2\pi } $” represents the proportion of a circle covered by the CT (*i.e.*, 0.106), and “$ \frac{1}{A} $” is the availability correction factor defined by the proportion of time when individuals of a given species are available for detection.

A precisely-determined availability correction factor is necessary to avoid important biases in abundance estimates (Cappelle et al., 2019). The Maxwell’s duiker is active mostly during the day, and at the same time is very abundant in TNP (22,101 videos). Therefore, we decided to include only data from the peak of activity between 7:00 and 7:59 (Howe et al., 2017; Cappelle et al., 2021) when the availability correction factor is 1. The Jentink’s duiker which is nocturnal species, was considered to be fully available for the CTs between 18:00 to 5:59 (correction factor equal 1) and only the data from this period were analysed (>90% videos). The survey duration was defined as 12 h 59 min 59 s.

The pygmy hippopotamus is a rather nocturnal species with 12% of videos during the day. It was not available for detection at all times because of resting in the shallows and rivers. Similarly, the chimpanzees, diurnal and semiarboreal species, were also not available for detection at all times at the day. Therefore, we defined “*T*_*k*_” as 24 h and estimated the proportion of time when pygmy hippopotamus and chimpanzees were available for detection using method described in Rowcliffe et al. (2014). We calculated the animal’s availability (A) using the r package “activity” (R Core Team, 2019) to determine the availability correction factor of these species.

We estimated the density using candidate models include the half-normal detection function with 0 and 1-hermite polynomial adjustment terms, the uniform detection function with 1 and 2-cosine adjustment terms and the hazard-rate detection function with 0, 1 and 2-cosine adjustment terms.

The best model was selected following a two-step procedure proposed by Howe et al. (2019). We selected the preferable models by comparing the adjusted model selection criteria (QAIC) scores within each key function. Then, we compared the goodness of fit test (GOF) statistic of the best models with different key function. We estimated variances using a nonparametric bootstrap, resampling points with replacement (Howe et al., 2017).

**Figure 2:**
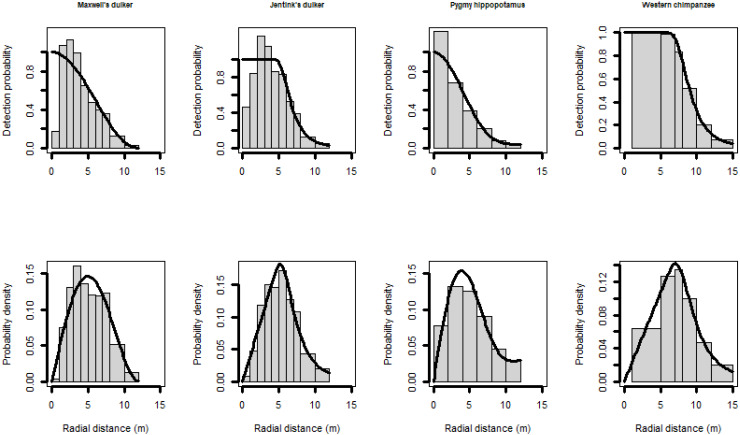
Scaled histogram of the detection probability and the probability density of Maxwell’s duiker, Jentink’s duiker, pygmy hippopotamus and Western chimpanzees as a function of distance. Reactive snapshots were excluded.

Following the exploratory analyses, we left-truncated the data when fewer detection near the CTs were recorded than expected, and right-truncated to eliminate the 10%–15% (approximately) of the farthest observations (Buckland et al., 2001). Therefore, longer distances were difficult to measure accurately, and we have very little data between 12–15 m (less than 1%) for Maxwell’s duiker, Jentink’s duiker and pygmy hippopotamus, either because of small size (Maxwell’s duiker) or a nocturnal behaviour (Jentink’s duiker and pygmy hippopotamus). Therefore, for these species we right-truncated distance observations > 12 m (Fig. 2). For pygmy hippopotamus, given its size, both body length and width were considered when measuring radial distances, so we combined distance observations into 2-m intervals. Most models of the detection function did not fit well for chimpanzees. In such cases, it is reasonable to left-truncate data for achieving accurate estimates (Buckland et al., 2001). We achieved a reasonable fit only when we left-truncated at 1-m and we combined distance observations of 1 to 5-m and 5 to 7-m (Fig. 2).

We finally estimated the population size by multiplying the estimated density by the study area size (5,360 km^2^), mentioned above.

### Factors influencing animal’s reaction to camera trap

We attempted to determine which variables influenced the animals’ reaction to CTs. We hypothesised that CTs could be detected by animals for the following reasons: (1) olfactory signals (metal, plastic and human odours on the device), (2) visual signals (neophobia towards foreign objects and infrared LED illumination by night) and (3) auditory signals (emission of sounds from the electronic and mechanical components of the device) (Séquin et al., 2003; Larrucea et al., 2007; Meek et al., 2014; Caravaggi et al., 2020).

To measure whether the animal reacts to the CTs due to olfactory signals, we used the variable “CT duration on site” (*i.e.*, the time elapsed between the date the CT was installed and the date the animal was recorded). The odour of the device itself or the odour left by the team on the device and on the nearby vegetation can remain for days. So, if animals react because of the odour of the device, then the effect should decrease the longer the CT stays in the field.

To determine whether the animal visually detected the CT, we used the distance to the CT (*i.e.*, the distance at which the animal was located from the CT) and the time of the day (*i.e.*, the time at which the reaction to the CT was observed). The time of the day was included for the species that were active at night (*i.e.*, the Jentink’s duiker and the pygmy hippopotamus). If animals reacted to the CT because they detected it visually, we supposed that the probability to react should decrease the further away the animal was from the CT. If animals reacted to the LEDs of the CTs, which were only visible at night, then the reaction level was higher by night. Unfortunately, we have not witnessed behaviour that were representative of auditory reaction (*e.g.*, freezing), so we did not analyse this factor.

We tested those variables using generalized linear models (GLMs) with binomial error structure and logit link function (McCullagh & Nelder, 1989) for each of the species. The three predictors were those described hereabove and the response was the reaction to CT (with reaction (1) or without reaction (0)). Since the time of the day is a circular variable, we transformed the time of the day into radian and included its sine and cosine into the model (Stolwijk, Straatman & Zielhuis, 1999).

Variance inflation factor (VIF; Field, 2005) were derived using the function VIF of the r-package “car” (Fox & Weisberg, 2019) applied to a standard linear model excluding the random effects. Thus, a variable is only retained in the model when its VIF is below the threshold value of 2.5 (Allison, 1999).

To establish the significance of the full model (Forstmeier & Schielzeth, 2011), we used a likelihood ratio test (Dobson, 2002) comparing its deviance with that of the null model comprising only the intercept. We considered a fixed effect being significant when the *P*-value is inferior to 0.05.

## Results

The CTs operated for a cumulative 24,637 camera-days. The analysis of 58,947 videos recorded enabled the identification of 98 different species (11 carnivores, 17 ungulates, eight primates, three pangolins, eight rodents and 49 birds), belonging to 79 genera, 42 families and 21 orders.

### Reaction to the camera trap

Maxwell’s duikers only showed attraction to CTs in 14 observations representing 0.11% of the sample (Table 3). On the contrary, Jentink’s duikers strongly reacted to the CTs. Obvious reactions of avoidance and attraction represented 19.25% of the observations, while the cases of inspection behaviour represented 13.60% of the observations *i.e.*, when the animal sniffed in all directions mainly towards the CT or sniffed branches cut or touched by field teams during CT installation (Table 1). Pygmy hippopotamus also strongly reacted to the CT but less visibly as 36.64% of the reactions were inspection behaviours whereas the obvious reactions constituted 16.31% of the data (Table 3). Finally, Western chimpanzees showed a similar rate of reaction to the pygmy hippopotamus regarding attraction and avoidance behaviour (15.23%) but a lower rate of inspection behaviour (1.17%) (Table 3).

**Table 3:** Proportion of reactions to the camera-trap device recorded every 2 s at predetermined snapshots via video clips of Maxwell’s duiker, Jentink’s duiker, pygmy hippopotamus and Western chimpanzee.

Behaviour type	Behaviour	Maxwell’s duiker; %; (N)	Jentink’s duiker; % (N)	Pygmy hippopotamus; % (N)	Western chimpanzee; % (N)
**Normal behaviour**	Moving/feeding/foraging	99.89%; (13,192)	67.16%; (2,450)	47.04%; (1,217)	83.60%; (2,421)
**Reaction to camera**	Attraction/fixation	0.11%; (14)	16.34%; (596)	12.37%; (320)	11.50%; (333)
	Inspection	0%; 0	13.60%; (496)	36.64%; (948)	1.17%; (34)
	Avoidance	0%; 0	2.91%; (106)	3.94%; (102)	3.73%; (108)
Total		13,206; 100%	100%; (3,648)	100%; (2,587)	100%; (2,896)

**Notes.**

(N) number of radial distances

After discarding all reactions to the CTs, the data including normal behaviour represented 99.89% of the total observations for the Maxwell’s duiker, 67.16% for the Jentink’s duiker, 47.04% for the pygmy hippopotamus and 83.60% for the Western chimpanzee (Table 3).

### Quantifying the bias in abundance estimate

Density estimates of Maxwell’s duikers indicated 21.19 ind./km^2^ keeping all reaction behaviours to CTs and 21.17 ind./km^2^ when excluding reaction behaviours. Hence there was a slight overestimation of the density of 0.02 ind./km^2^ (107 individuals).

Concerning the Jentink’s duikers, the analysis of all data including reactions provided an estimate of 0.75 ind./km^2^. The density decreased to 0.68 ind./km^2^ when excluding only attraction and avoidance behaviour and dropped to 0.26 ind./km^2^ when excluding all reactions. Therefore, if we had kept the implicit reactions, we would have overestimated the density of 0.46 ind./km^2^. Similarly, regarding the pygmy hippopotamus, the determined availability was 49% over a 24-hour period. The density estimate was 0.81 ind./km^2^ including all reactions, 0.59 ind./km^2^ when excluding the attraction and avoidance of the CT and 0.23 ind./km^2^ when excluding all reactions.

Finally, the chimpanzees were available for detection 33% of the time every day. Using the availability correction factor, we found a density of 0.38 ind./km^2^ when considering all reactions, 0.26 ind./km^2^ without obvious reactions and 0.23 ind./km^2^ excluding all reactions. We observed an overestimation of density of 0.15 ind./km^2^ between analyses that included all reactions to CTs and those that excluded all reaction behaviours to CTs.

The abundance estimates remained precise for Maxwell’s duikers with a coefficient of variation (CV) of 10% for the three analyses while an increasing of CV was observed for the other three species, particularly for Western chimpanzees for which the CV increased from 30% to 43% (Table 2).

### Factors influencing animal’s reaction to camera trap

The VIF of each of the different explanatory variables was less than 2.5, *i.e.*, the threshold value. All the full-null model comparison of the four species were significant (Maxwell’s duiker: *χ*^2^ = 47.02, *p* < 0.001; Jentink’s duikers: *χ*^2^ = 1032.53, *p* < 0.001; pygmy hippopotamus: *χ*^2^ = 1110.42, *p* < 0.001; Western chimpanzee: *χ*^2^ = 509.67, *p* < 0.001).

**Figure 3:**
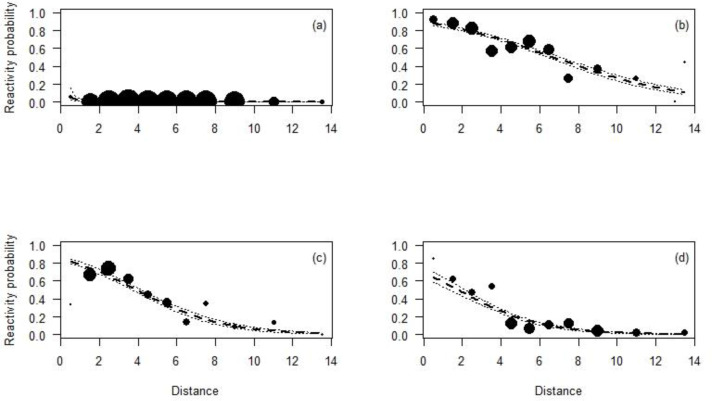
Reactivity probability to the camera as a function of distance. (A) Maxwell’s duiker; (B) Jentink’s duiker; (C) pygmy hippopotamus and (D) Western chimpanzee. Black dots of different sizes represent sample size.

All species reacted less to the CTs at longer distances than closer ones (Table 4; Fig. 3). In fact, Maxwell’s duikers only reacted when located in the first meter from the CT and with a probability of reaction of less than 10%. On the contrary, the probability to react close to the CT was higher for the Jentink’s duiker, pygmy hippopotamus and Western chimpanzees with 90%, 80% and 65% respectively. Western chimpanzees reacted mainly within 5 m from the CT, while the pygmy hippopotamus and the Jentink’s duiker showed reaction even farther away (beyond 5 m), with probability of reaction slowly decreasing with distance from the CTs (Fig. 3).

**Table 4:** Results of the generalized linear models (GLMs) for test influence of olfactory and visual signals on the animals’ reaction to camera. Estimates and their standard errors (SE) from the full model, results of likelihood ratio tests (*χ*^2^) comparing the full model with a reduced model lacking the relevant term, degrees of freedom (df), the *p* values from the likelihood ratio test and confidence intervals (CIs).

Species	Parameters	Estimate ± SE	*z* value	*χ* ^2^	df	*p*	CIs	
							**2.5%**	**97.5%**
Maxwell’s Duiker	(Intercept)	−1.343 ± 0.93	−1.437	_	_	_	−3.350	0.341
	Distance estimated	−2.784 ± 0.59	−4.714	46.53	1	<0.001	−4.034	−1.707
	Time observation	4.297e−04 ± 0.008	0.056	0.003	1	0.96	−0.016	−0.015
Jentink’s Duiker	(Intercept)	1.358 ± 0.106	12.759	_	_	_	1.151	1.568
	Distance estimated	−0.565 ± 0.022	−25.767	1008.35	1	<0.001	−0.609	−0.523
	Time observation	0.003 ± 0.001	2.331	5.41	1	0.02	4.888e−04	0.006
	sin(time)	−0.405 ± 0.046	−8.819	83.7	2	<0.001	−0.430	−0.380
	cos(time)	−0.051 ± 0.096	−0.533		2	0.594	−0.076	−0.026
Pygmy Hippopotamus	(Intercept)	3.113 ± 0.127	24.380	_	_	_	2.867	3.367
	Distance estimated	−0.440 ± 0.024	−18.600	488.31	1	<0.001	−0.487	−0.394
	Time observation	−0.047 ± 0.002	−20.310	662.91	1	<0.001	−0.052	−0.043
	sin(time)	0.022 ± 0.077	0.291	29.7	2	0.771	−0.129	0.174
	cos(time)	−0.598 ± 0.116	−5.162		2	<0.001	−0.828	−0.374
Western Chimpanzee	(Intercept)	1.354 ± 0.156	8.700	_	_	_	1.052	1.663
	Distance estimated	−0.463 ± 0.03	−17.456	439.22	1	<0.001	−0.516	−0.412
	Time observation	−0.013 ± 0.002	−5.548	32.40	1	<0.001	−0.018	−0.008

**Figure 4:**
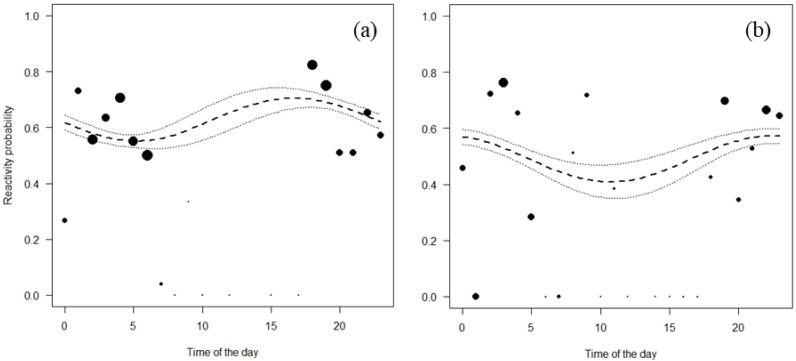
Reactivity probability to the camera as a function of time of the day. (A) Jentink’s duiker and (B) pygmy hippopotamus. Black dots of different sizes represent sample size.

Both Jentink’s duikers and pygmy hippopotamus reacted significantly more at night than during the day. All the full-null model comparison of Jentink’s duikers and pygmy hippopotamus were significant (Jentink’s duikers: *χ*^2^ = 83.7, *p* < 0.001; pygmy hippopotamus: *χ*^2^ = 29.7, *p* < 0.001) (Table 4; Fig. 4).

**Figure 5:**
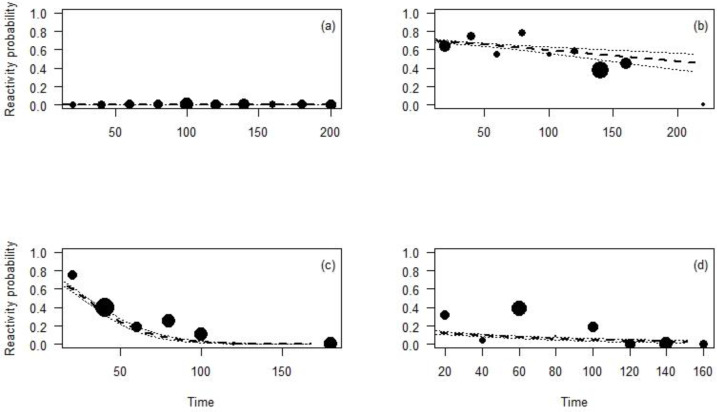
Reactivity probability to the camera as a function of the time between installation of the camera and observation moment. (A) Maxwell’s duiker; (B) Jentink’s duiker; (C) pygmy hippopotamus and (D) Western chimpanzee. Black dots of different sizes represent sample size.

The effect of the time CTs were on the field on the animal reaction was significant for all the species except for the Maxwell’s duiker (*p* = 0.96; Table 4; Fig. 5) and differed depending on the species. During the first days the CT was set up, the reaction probability for the Jentink’s duiker remained high, 70% of observations but decreased slightly over time. The reaction probability observed for the pygmy hippopotamus was 65% during the first days the CT was set up and slowly decreased until after 100 days from CT installation. The probability of reaction of Western chimpanzees remained low (<20%) despite a significant decrease with the time (Fig. 5).

## Discussion

This study is based on CTDS method applied to multiple species and over a broad geographic scale. Our results demonstrated clearly that using the species-specific ethograms allowed to detect the different ways and levels each species reacted to CTs. As in other CTs studies (Meek et al., 2014; Caravaggi et al., 2020), we found that external factors influenced the behaviour of the animals towards the CTs such as olfactory and visual signals. In this case, we went further in the analysis of the animals’ behaviours especially for implicit behaviours (inspection reaction).

The Maxwell’s duiker is one of the most abundant and best distributed species in TNP with 21.17 ind./km^2^. In 2011, Hoppe-Dominik et al. reported a density of 17.1 ind./km^2^ in the same area using a different method. More recently, Howe et al. (2017) and Cappelle et al. (2021) respectively reported 14.5 ind./km^2^ and 19.74 ind./km^2^ in the research area of the TNP using the CTDS method. Even though our estimates are higher than in other studies, they show overlapping confidence intervals with a precise coefficient of variation of 10%. Additionally, the Maxwell’s duiker is abundant and well distributed everywhere which also contributed to the precision of the result by giving us a total estimate of 113,468 individuals in the TNP. The high number is consistent with the ecology of the species. Indeed, Maxwell’s duikers have broad and adaptable feeding habits, allowing them to find sufficient food reserves in primary and secondary forests as well as in cleared areas (Happold, 1987; Hofmann & Roth, 2000). Moreover, they are a fast-reproducing species, with average age at sexual or reproductive maturity about 18 months with a gestation period is about 120 days. The female breed once a year and give birth to single young (Kingdon, 1974; Happold, 1987).

The Jentink’s duiker is endemic to the western part of the Upper Guinea Forests, from Sierra Leone to western Côte d’Ivoire (Hoppe-Dominik, 2013), and is relatively unknown due to its rarity, its secretive nature and the fact it is confined to remote primary forests, although it may use secondary forests on a seasonal basis (Davies & Birkenhäger, 1990; Newing, 2001). Here, we estimated 0.26 ind./km^2^ (1,389 individuals) with reasonable precision (CV=18%), which provides the first estimates of Jentink’s duikers, an understudied species in the TNP. Other studies estimated the density on its range-wide basis of 0.1 ind./km^2^ (East, 1999), but it can reach 1 ind./km^2^ in well-preserved areas (Peal & Kranz, 1990). The world population is estimated at about 3,500 individuals (East, 1999), meaning that TNP would harbour more than 1/3 of the existent individuals. However, the world population estimate might be underestimated as they are based on past biomonitoring methods, such as distance sampling with line transects that might fail to monitor elusive and shy species since species flee before being detected. It violates distance’ assumptions and creates a negative bias in estimates (Buckland et al., 2001). The Jentink’s duiker reacted strongly to the CTs in visual and olfactive ways. It was observed on a few videos that the Jentink’s duiker tended to be attracted to the odour left by human contact on and around the CTs, and to rub against the same points. In addition, we noted that this attraction lasted for several months. This observed behavioural may be due to this species being sedentary and territorial (Kingdon, 1997).

The distribution area of the pygmy hippopotamus is the same as for the Jentink’s duiker and the population estimate is 2,000–3,000 individuals (Mallon et al., 2011; Ransom, Robinson & Collen, 2015) over the whole range. We estimated the pygmy hippopotamus population at 0.23 ind./km^2^, a first for the TNP, resulting in 1,255 individuals living in the TNP. Other studies have already suggested that the TNP would harbour the largest wild population of the species (Roth et al., 2004). Also, their presence can be explained by the abundance of rivers in the park as it is known to live near marshy areas (presence of water and moist soil), in shallows and rivers (Bogui et al., 2016; Ouattara et al., 2018). Among the four species, pygmy hippopotamus is the one that reacted the most to the CTs and more than half of the data had to be deleted for the abundance determination as they often showed inspection behaviours. As a consequence, more observations than expected were recorded between 0 and 2 m from the CTs. In the 50 days following the installation of the CTs, the reaction probability to the CT remained approximately at 30% of the video captures. Similarly, when the animal was 4 m from the CTs, the reaction probability was still 50%. Such a situation could be due to the CTs which according to Kalan et al. (2019) would represent a new visual and chemical element in those animals’ environment towards with individuals being intrinsically more neophilic or neophobic. Moreover, it is possible that human scents on device and on trees and branches affect some species or individuals’ behaviour more than others (Caravaggi et al., 2020), such as observed for pygmy hippopotamus. The attraction to CTs may be exacerbated by clearing immediate vegetation to reduce false triggers, potentially leaving behind additional cues of recent human presence (Caravaggi et al., 2020). Furthermore, it is possible that pygmy hippopotamus is able to see the infrared illumination used by CTs. Meek et al. (2014) showed that the infrared illumination produced by CTs may be undetectable by humans but that many animals were able to see it. In Canada, studies found 40% of wolves (*Canis lupus*) showed an adverse response to infrared CTs (Gibeau & McTavish, 2009).

The Western chimpanzees’ population is well documented for the TNP since the mid-70s based on regular monitoring by line transects (Hoppe-Dominik et al., 2011) and the population was estimated to be in average 404 individuals (CI [240–687]) with an average %CV of 28% from the period 2005-2020. Through this new study, we found 1,236 individuals (CI [472–2504]) with a %CV of 43%. This new estimate is larger than any obtained annually with the previous method. The line transect method is known to underestimate the chimpanzees’ population due to the difficulties for the observers to detect chimpanzees’ nests in the canopy (Plumptre & Reynolds, 1996; Buckland et al., 2001; Walsh & White, 2005; Cappelle et al., 2021). This new estimate is also very imprecise: first because the chimpanzees were filmed by only 24% of CTs, second because two different locations accumulated 48.6% of the observations, as large fruiting trees attracted many chimpanzees feeding for long duration at different time without any reaction to the CTs. The variability in the number of observations between points transect increases the variance of estimates. To avoid this problem, Cappelle et al. (2019) suggested to increase the number of CTs.

Our results showed that animals reacted differently to CTs. Not taking into account animals’ specific-reaction to CTs could led to an underestimation or an overestimation of the density estimates (Bessone et al., 2020). For species with insufficient data substantially more CTs would be required to improve the accuracy of the density (Cappelle et al., 2021).

When using the CTDS method, we recommend to use the same model of CTs in order to reduce the effect of infrared illumination, preferably the LEDs no glow CTs (black LEDs) which do not emit light when triggered. It is important to not only use the same model of CTs, but to make sure that all CTs are operated on the same settings, the same installation and set-up in order to provide the most consistent, robust, and reproducible data possible. In order to minimise the bias due to behavioural responses, we recommend to adopt a set of measures on the field and in the data analyses. In the field, we recommend to reduce the number of researchers on site during the CTs installation to limit the environment contamination by human odour, and/or to deploy CTs at least 1 month prior to the survey to enable animals getting used to CTs, and/or to record reference distance labels after the survey to limit the time of CTs installation and the presence of human odour on device and in the nearby environment (Caravaggi et al., 2020; Bessone et al., 2020). If all these recommendations cannot be applied, other methods that are not influenced by reaction to CTs (*e.g.*, SECR), could be used instead of CTDS. When analysing the data for density and abundance estimates, we also recommend to carefully check species-specific reactions in videos and exclude them from the analyses.

## Conclusions

The application of CTDS to diverse species enabled us to provide for the first density estimates of understudied species. In addition, we showed that adopting a very clear species-specific ethogram could clearly lead to a more objective population density and abundance estimates. Thus, excluding all reactions from the analysis can provide more reliable abundance estimates of multiple species to inform conservation decisions and better assess the status and trends of animals’ population. Repeating this monitoring survey over several years will allow population trends to be assessed and crucial information for wildlife conservation to be provided.

## Supplemental Information

10.7717/peerj.13510/supp-1 Supplemental Information 1 Dataset for Maxwell’s duiker, Jentink’s duiker, Pygmy hippopotamus and Western chimpanzee

## Additional Information and Declarations

### Data Availability

The following information was supplied regarding data availability: The raw data is available in the Supplemental File.
